# Flower-Like Internal Emission Distribution of LEDs with Monolithic Integration of InGaN-based Quantum Wells Emitting Narrow Blue, Green, and Red Spectra

**DOI:** 10.1038/s41598-017-07808-2

**Published:** 2017-08-02

**Authors:** Kwanjae Lee, Ilgyu Choi, Cheul-Ro Lee, Tae-Hoon Chung, Yoon Seok Kim, Kwang-Un Jeong, Dong Chul Chung, Jin Soo Kim

**Affiliations:** 1 Division of Advanced Materials Engineering & Research Center of Advanced Materials Development, Chonbuk National University, Jeonju, 54896 Republic of Korea; 2 Korea Photonics Technology Institute, Gwangju, 61007 Republic of Korea; 3 Department of Nano-Optical Engineering, Korea Polytechnic University, Siheung, 15073 Republic of Korea; 4 Department of Polymer-Nano Science and Technology, and Polymer Materials Fusion Research Centre, Chonbuk National University, Jeonju, 54896 Republic of Korea; 5 Korea Institute of Carbon Convergence Technology, Jeonju, 54853 Republic of Korea

## Abstract

We report a phosphor-free white light-emitting diodes (LED) realized by the monolithic integration of In_0.18_Ga_0.82_N/GaN (438 nm, blue), In_0.26_Ga_0.74_N/GaN (513 nm, green), and In_0.45_Ga_0.55_N/In_0.13_Ga_0.87_N (602 nm, red) quantum wells (QWs) as an active medium. The QWs corresponding to blue and green light were grown using a conventional growth mode. For the red spectral emission, five-stacked In_0.45_Ga_0.55_N/In_0.13_Ga_0.87_N QWs were realized by the *so-called* Ga-flow-interruption (Ga-FI) technique, wherein the Ga supply was periodically interrupted during the deposition of In_0.3_Ga_0.7_N to form an In_0.45_Ga_0.55_N well. The vertical and lateral distributions of the three different light emissions were investigated by fluorescence microscope (FM) images. The FM image measured at a focal point in the middle of the n-GaN cladding layer for the red-emitting LED shows that light emissions with flower-like patterns with six petals are periodically observed. The chromaticity coordinates of the electroluminescence spectrum for the white LEDs at an injection current of 80 mA are measured to be (0.316, 0.312), which is close to ideal white light. In contrast with phosphor-free white-light-emitting devices based on nanostructures, our white light device exhibits a mixture of three independent wavelengths by monolithically grown InGaN-based QWs, thus demonstrating a more facile technique to obtain white LEDs.

## Introduction

Generally, the most common technology to fabricate white light-emitting diodes (WLEDs) is to combine a phosphor-wavelength converter with blue or ultraviolet III-nitride chips. However, phosphor materials degrade during long-term optical pumping, resulting in the significantly decreased output efficiency of phosphor-based WLEDs. In addition, since the light emitted by phosphor-based WLEDs is usually composed of two-color spectra such as blue and yellow, a color rendering index (CRI) lower than that of ideal white light is inevitable^1, 2^. To solve this problem, phosphor-based WLEDs with a mixture of blue, green, and red phosphors with a UV-LED chip were recently fabricated. However, many problems remain, such as the low efficiency of the red phosphors and the need for complex coating technique^3^, and their fabrication requires a complicated packaging process. Therefore, in terms of device performance, fabrication processes, and cost, it is preferable to realize WLEDs without phosphor materials by monolithically growing three different sets of III-nitride quantum structures corresponding to blue, green, and red light. Recently, III-nitride-based nanostructures such as quantum dots and nanowires have been investigated to extend emission wavelengths to long-visible wavelengths, including orange and red spectral ranges, especially for the fabrication of white light sources. Although III-nitride nanostructures offer significant advantages over conventional LEDs with InGaN/GaN quantum wells (QWs), such as fewer threading dislocations and a reduced quantum-confined Stark effect (QCSE), the device performance suffers severely from the large amount of surface states and defects^4, 5^. As a result, LEDs with nanostructures as the active media still exhibit relatively low output power, often less than a few microwatts^6^. In addition, commercial blue or green LEDs are based on InGaN/GaN QW structures, and thus, obtaining long-visible wavelengths over ~550 nm using QWs is a significant achievement. However, the realization of wavelengths above ~550 nm has been limited, mainly because of difficulties in growing high-quality InGaN/GaN QWs due to the large difference between the lattice constants, thermal expansion coefficients, and optimal growth conditions of InGaN and GaN^7–10^. In particular, it is difficult to obtain InGaN with a high In content (HI-InGaN) applicable to an active layer emitting yellow-red wavelength; thus, fabricating long-wavelength LEDs with high efficiency has been relatively more difficult than fabricating blue and green LEDs^11^. Therefore, it is important to improve efficiency of yellow-red QWs to realize high-performance WLEDs. S. Saito *et al*. reported an improvement in the emission intensity of yellow-red LEDs by engineering the energy band using two-step high-temperature growth and an AlGaN interlayer^12^. K. Ohkawa *et al*. introduced a special metal-organic vapor-phase epitaxy technique using a micro-flow channel to increase the In-content of InGaN/GaN QWs^13^. In addition, yellow-red LEDs have been reported using non-polar or semi-polar substrates^14–16^. Although phosphor-free WLEDs with two or three emission wavelengths have been realized using InGaN/GaN multiple QWs (MQWs) with different In contents^17, 18^, the blue, green, and red emissions were not clearly distinguished in their electroluminescence (EL) spectra.

In this work, we successfully realized phosphor-free WLEDs with monolithically grown InGaN-based MQWs with clearly distinguished blue, green, and red spectra. InGaN/GaN MQWs with blue and green emissions were grown using a conventional growth method, wherein the In, Ga, and N sources were simultaneously supplied to grow the InGaN wells. For the red emission, In_0.45_Ga_0.55_N/In_0.13_Ga_0.87_N QWs were prepared, wherein the In_0.45_Ga_0.55_N wells were realized by the *so-called* Ga-flow-interruption (Ga-FI) technique. The optical and electrical properties of the LED samples were studied via their photoluminescence (PL) and EL spectra. To quantitatively investigate the In contents of the MQWs, cross-sectional transmission electron microscope (TEM) images were recorded in the color-coded mode (CCM). In addition, the vertical and lateral distributions of the samples’ luminescence were investigated using fluorescence microscope (FM) images measured by changing the focal point (FP).

## Results and Discussion

**Figure 1:**
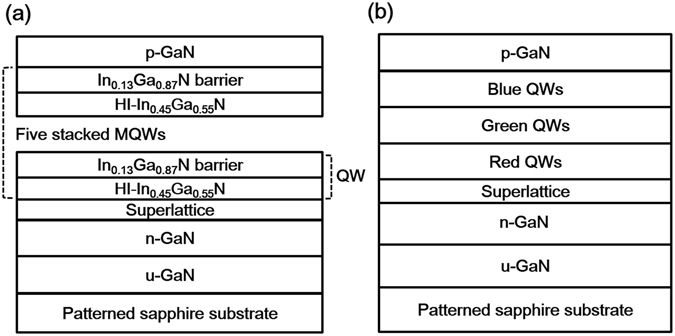
Schematic structures of (**a**) the RLED and (**b**) WLED.

Figure 1(a and b) show the schematic structures of a red-emitting LED (RLED) and a WLED, respectively. For the WLED, three different sets of MQWs, namely, blue, green, and red QWs were monolithically grown on a patterned sapphire substrate (PSS) by Thomas–Swan metal-organic chemical-vapor deposition (MOCVD). The active region consists of two stacks of blue In_0.18_Ga_0.82_N/GaN QWs, three stacks of green In_0.26_Ga_0.74_N/GaN QWs, and five stacks of red In_0.45_Ga_0.55_N/In_0.13_Ga_0.87_N QWs. The In_0.45_G_0.55_N layers of the red QWs were grown by the Ga-FI technique, wherein the Ga supply was periodically interrupted during the deposition of In_0.3_Ga_0.7_N. Further details on the LED structures and growth conditions can be found in the Experimental Section.

**Figure 2:**
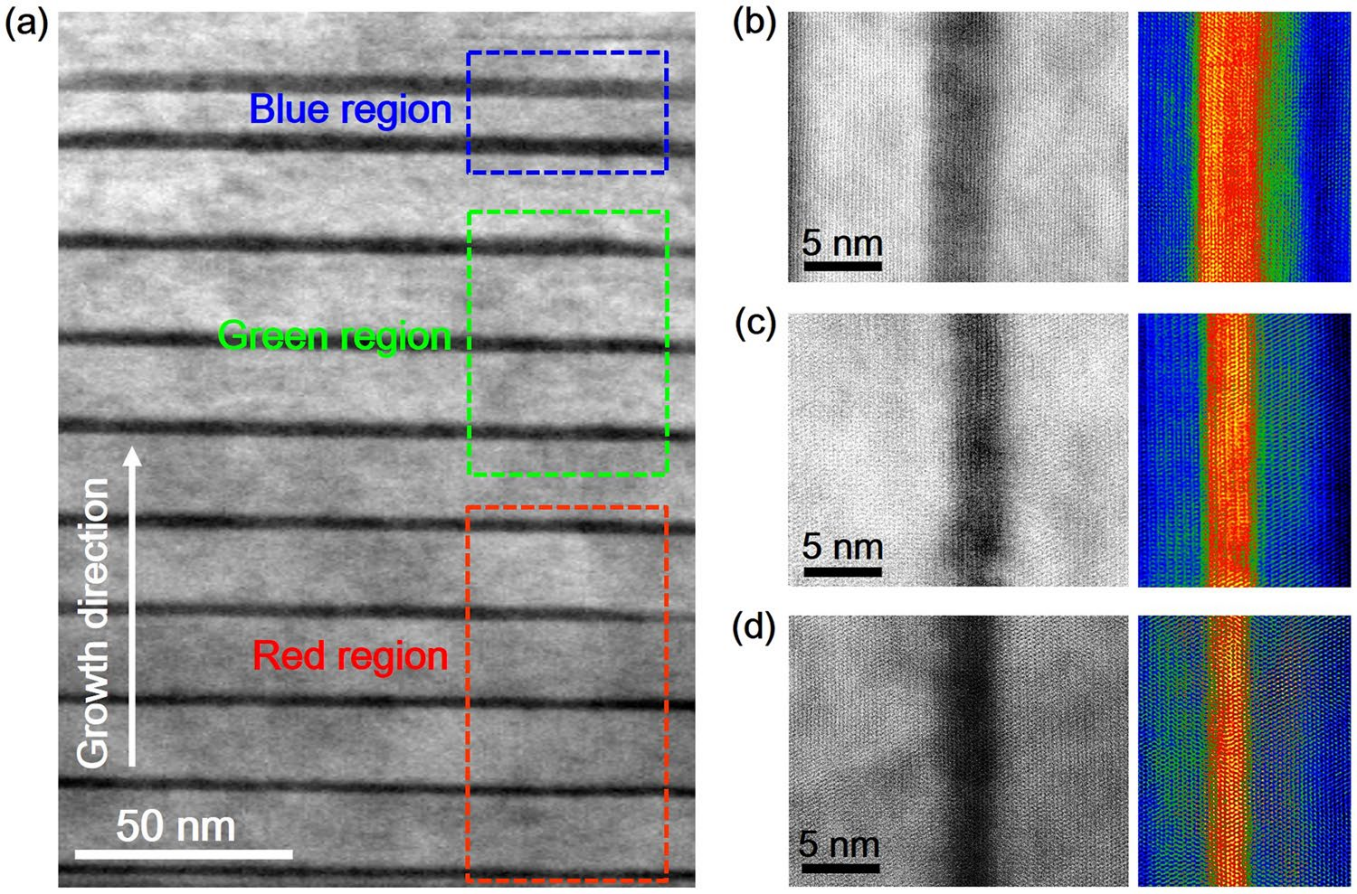
(**a**) Cross-sectional TEM image of the WLED with blue, green, and red QWs. (Left) STEM and (right) CCM-TEM images of the (**b**) blue, (**c**) green, and (**d**) red QWs, wherein red and blue indicate high and low Ga:In ratios, respectively.

Figure 2(a) shows a cross-sectional TEM image of the WLED sample, wherein the three different sets of MQWs are distinguished by dashed squares. The average thicknesses of the InGaN well (barrier) for the blue, green, and red QWs were measured to be 3.93 nm (7.97 nm), 3.08 nm (17.44 nm), and 2.61 nm (16.77 nm) nm, respectively. Usually, the crystal quality of InGaN grown by the conventional growth mode degrades with the increasing In content, mainly due to the complex growth kinetics of In and Ga adatoms. In previous reports, determining the interface between the HI-InGaN well and the GaN barrier was difficult for HI-InGaN/GaN QWs grown by the conventional growth mode. In addition, many In-rich clusters were generated in the HI-InGaN well^19^. However, in this work, the interface between the In_0.45_Ga_0.55_N well and the In_0.13_Ga_0.87_N barrier for the In_0.45_Ga_0.55_N/In_0.13_Ga_0.87_N QWs is relatively more distinct and higher quality than those of the previous results^20, 21^. The improvement of interface quality between the In_0.45_Ga_0.55_N well and the In_0.13_Ga_0.87_N barrier for the RLED, preparared by the Ga-FI technique, is related to the increase in possiblity for In adatoms to migrate toward their optimal positions. Figure 2(b,c and d) show scanning TEM (STEM) images of a single QW from the blue, green, and red active regions, respectively. More In-rich clusters were observed in the QW with the increasing In content in the InGaN due to its intrinsic phase separation behavior, which was largely caused by the miscibility gap between InN and GaN^22^. To visualize the compositional distribution at the wells and barriers of the WLED sample, CCM-TEM images, wherein the colors provide information on the In and Ga contents, are displayed to the right of each STEM image. Specifically, vibrant red and blue regions correspond to a designed InGaN well and a barrier, respectively. Green regions with different contrast below and above the InGaN well were due to the different content ratio of In and Ga atoms, which is related to the well-known inter-diffusion phenomenon of In and Ga atoms at the well/barrier interface. For the blue and green QWs, a thin green line appeared at the bottom of the InGaN well. However, indistinct green regions were spatially distributed in the barrier above the well over a relatively large area. This can be explained by the diffusion of In atoms from the InGaN well to the GaN barrier, which is largely due to the high growth temperature for the GaN barrier. In the CCM-TEM image of the red QW shown in Fig. 2(d), the green region is more indistinct over a large area than in those of the blue and green QWs. This can be attributed to an increase in the inter-diffusion of In and Ga atoms at the interface between the well and the barrier because of higher In content in the InGaN well. Furthermore, the red region is thinner and less vibrant than in the other images, indicating the higher In content in the red QW barrier than in those of the blue and green QWs because In_0.13_Ga_0.87_N was used as a barrier for the red QW.

**Figure 3:**
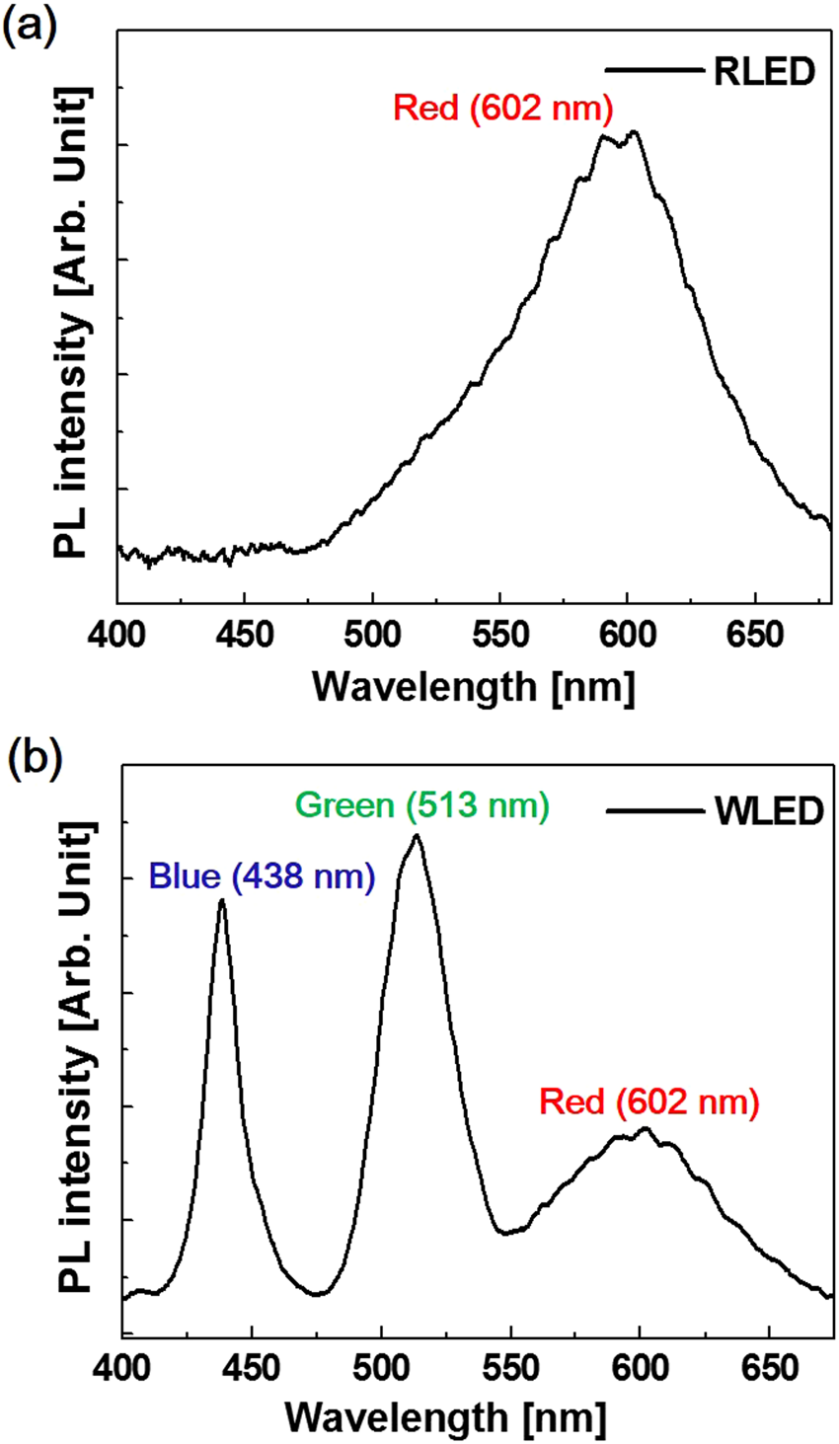
PL spectra of (**a**) the RLED and (**b**) WLED measured at RT.

Figure 3(a) shows the PL spectrum of the RLED sample measured at room temperature (RT). The wavelength and full-width at half-maximum (FWHM) of the peak were measured to be 602 nm and 76 nm, respectively. Figure 3(b) shows the PL spectrum of the WLED sample, where the peak wavelengths were measured to be 438 nm, 513 nm, and 602 nm, respectively corresponding to blue, green, and red lights. The FWHMs for the blue, green, and red peaks were measured to be 17.5 nm, 30.7 nm, and 73 nm, respectively. The FWHM of the red peak is wider than those of the blue and green peaks, which can be explained by more In segregation in the In_0.45_Ga_0.55_N/In_0.13_Ga_0.77_N QWs. In general, compositional uniformity in InGaN degrades with increasing In content largely due to the different local migration behaviors of In and Ga atoms^23, 24^. The internal quantum efficiency (IQE) of the WLED was 47.43%, which was evaluated from the integrated PL intensities measured at 10 K and RT [Figure S1 at “Supplementary information” shows the PL spectra for the WLED measured at 10 K and RT]. The PL intensity of the red emission was lower than those of the blue and green emissions. However, it is noteworthy that the intensity of the red emission could be plotted on the same linear scale as that of the blue and green emissions. Even though the stacking number of red QWs is more than those of blue and green QWs, this is a meaningful result. That is, this was not possible in most of the previous works. The lower red PL intensity can be explained by the increase in the QCSE due to the high In content in the red QWs. In other words, the increase in the QCSE resulted in the reduction of the overlap integral between the electron and hole wave-functions in the QW region. In addition, the increase in the fluctuations of localized states and the degradation of the crystallinity with the increasing In content in InGaN are responsible for the lower red PL intensity.

**Figure 4:**
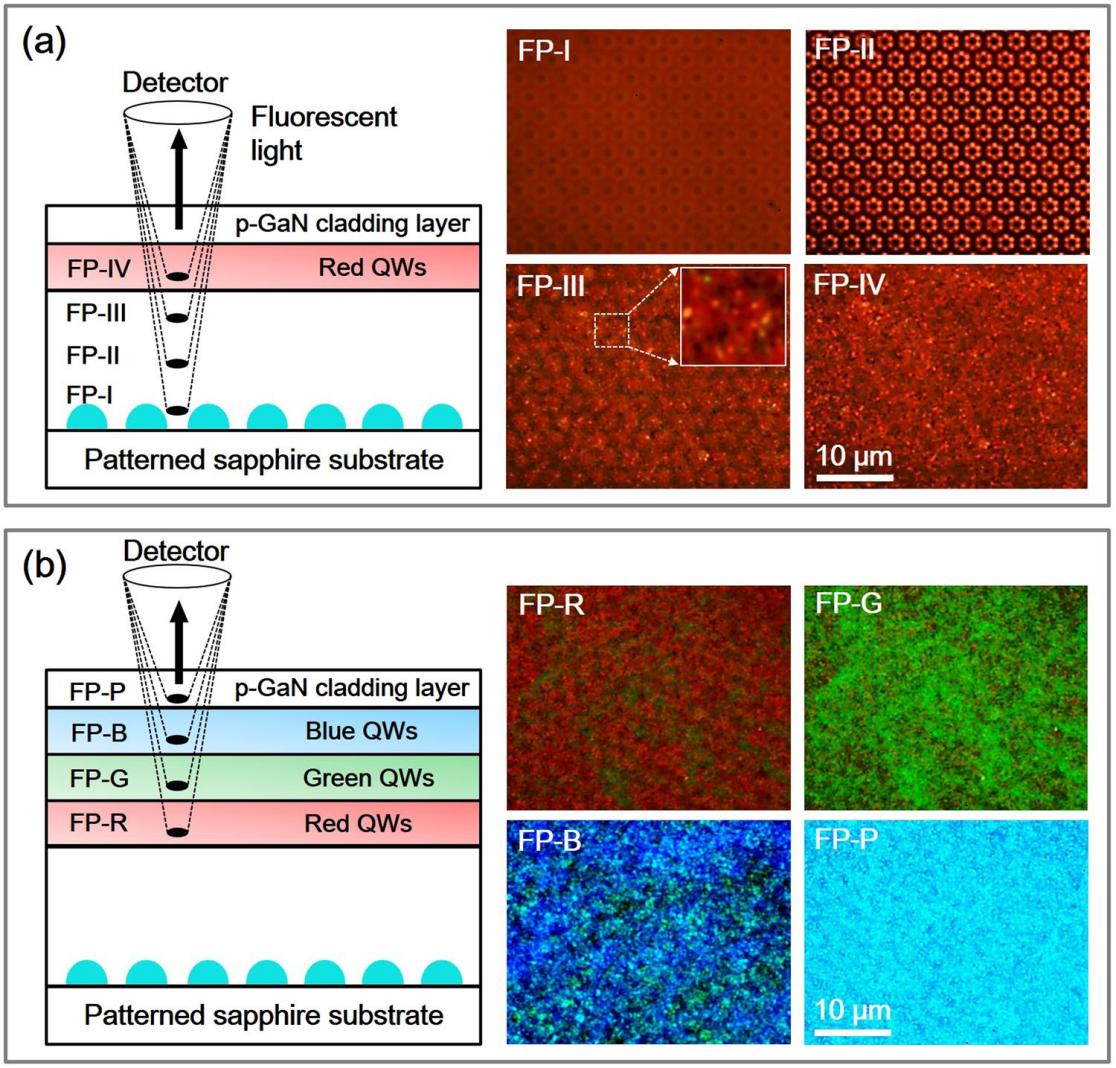
(**a**) Schematic diagram of the four different FPs positioned in the vertical direction of the RLED for FM measurements and the FM images obtained from the FPs of FP-I, FP-II, FP-III (the inset is a magnified view), and FP-IV. (**b**) Schematic diagram of the four different FPs positioned in the vertical direction of the WLED and the FM images obtained from the FPs of FP-R, FP-G, FP-B, and FP-P.

To analyze the vertical and lateral distribution of light emission as a function of the In composition of the InGaN LED samples fabricated on the PSS with lens-shaped patterns (LSPs), FM measurements were carried out. Figure 4(a) shows a schematic diagram with four different FPs in the vertical direction of the RLED for the FM measurements. The right side of Fig. 4(a) shows FM images obtained from the RLED at various FPs, namely FP-I, FP-II, FP-III, and FP-IV. At FP-I, dark circles corresponding to the LSPs of the PSS were periodically observed. The red image among the LSPs is due to the downward light from the red QWs and the beam reflected from the interface between the PSS and the GaN epitaxial layer. For FP-II, corresponding to the middle of the n-GaN cladding layer, emission was mostly observed around the LSPs. The light emission took the shape of a flower with six petals. It is noteworthy that the flower-like emission images were also observed for the LEDs emitting violet, blue, cyan, green, yellow, and red colors [Figure S2 at “Supplementary information” shows the FM images of the LED samples measured in the middle of the n-GaN cladding layer]. This unique feature in the light distribution can be explained by interference effects due to the periodically positioned LSPs. The distance between the periodic bright features (*y*_bright_) around each LSP can be simply calculated using Young’s equation, *y*_bright_ = *L*(*m*λ/*d*)^25^, where *L* is the length from a screen with slits to the viewing screen, *m* the order number, λ the light wavelength, and *d* the distance between the slits. In this work, the inter-slit distance *d* between the LSPs and the length *L* were 1 and 1.3 µm, respectively. A simple calculation for the RLED with an emission wavelength of 596 nm, obtained from the PL measurement, indicated a distance of 774 nm between the adjacent bright patterns. In the FM image of the RLED from FP-II in Fig. 4(a), the distance between adjacent bright patterns was measured to be 775 nm, which agrees well with the calculation. The center regions of the LSPs were relatively dark, indicating low emission, which is related to the behavior of threading dislocations. In previous works, these dislocations initially formed at the boundary of the patterns, i.e., the interface between the patterns and the planar region^26, 27^. The dislocations then converged from the boundary to the top of the pattern region by staircase-upward propagation. As a result, the dislocations were mostly observed above the top region of the pattern, and the numerous dislocations propagated along the c-axis direction due to the vertical growth mode^25–31^. For FP-III, which is right below the QWs, red emission was mostly observed with small dark spots corresponding to the LSPs. In addition, small green, yellow, and orange spots were randomly observed, indicating spatial fluctuations in the In content of the In_0.45_Ga_0.55_N/In_0.13_Ga_0.87_N QWs. For FP-IV of the QWs, red emission was mostly observed with randomly distributed dark features, but the periodic interference from the LSPs was no longer observed. Figure 4(b) shows FM images for the WLED obtained at different FPs corresponding to the red (FP-R), blue (FP-B), and green (FP-G) QW regions, and the p-GaN cladding layer (FP-P). Similar to the FP-IV images from the RLED, the information on the LSPs was not observed in the FM images of the WLED. For FP-R corresponding to the red-QWs, red light was mostly observed. However, there are blue and green features originating from the blue and green QWs and the reflected beam from the interface between the PSS and the GaN cladding layer. Most of the emission in the FM image obtained at FP-G is green, with some red and dark spots. At FP-B, blue, green, cyan, and dark spots appear due to the existence of three different colors and dislocations/defects. At the highest FP (FP-P), light cyan was observed because of the mixture of the three colors.

**Figure 5:**
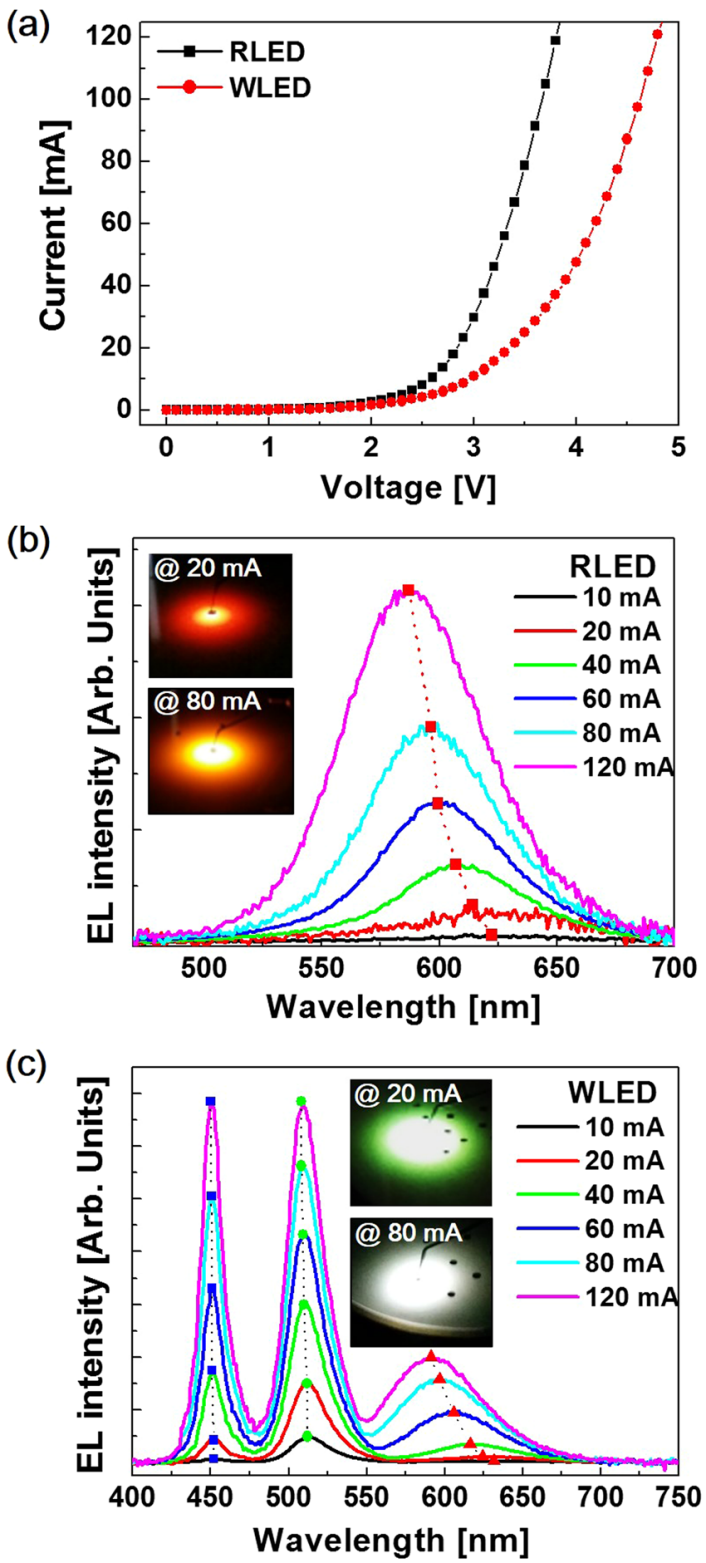
(**a**) I–V curves of the LED samples and EL spectra of the (**b**) RLED and (**c**) WLED at injection currents ranging from 10 to 120 mA. The insets are emission images taken during the EL measurements at injection currents of 20 (top) and 80 mA (bottom).

Figure 5(a) shows the current-voltage (I-V) characteristic curves of the LED samples. At an injection current of 20 mA, the forward voltages for the RLED and the WLED were 2.85 and 3.36 V, respectively. Figure 5(b and c) show the EL spectra of the two LED samples with the injection current increasing from 10 to 120 mA, and the insets of each spectrum show luminescent images taken at injection currents of 20 and 80 mA. In Fig. 5(b), the EL peak wavelength of the RLED sample blue-shifts with increasing injection current, which is attributed to the band-filling effect and partial compensation of the QCSE^32, 33^. For the RLED sample, the degree of blue-shifting at currents ranging from 10 to 120 mA was measured to be 35 nm. At an injection current of 20 mA, the luminescence of the RLED sample was orange-red. The peak wavelength at 80 mA was 596 nm, which appears yellow-orange. Figure 5(c) shows the EL spectra of the WLED with increasing injection current, which exhibits three clear emission peaks positioned at 448, 510, and 597 nm at an injection current of 80 mA. Bright white light was emitted due to the mixture of these three primary colors. Typically, the color purity of phosphor-free WLEDs using InGaN-based MQWs is strongly depending on the contribution degree of the red emssion. In the EL spectra of the WLED, the red emission could be plotted on the same linear scale as that of the blue and green emissions. This result indicates that the Ga-FI growth technique suggested in this work can be an effective way to extend emission wavelength to yellow-red spectral region with less degradation of radiative efficiency. As a restult, we fabricated phosphor-free WLEDs with high color purity. The chromaticity coordinates (Commission Internationale de l’Éclairage 1931, CIE) of the WLED sample were measured to be (0.316, 0.312), which is very close to the ideal white-light source of (1/3, 1/3). At this time, the CRI of the WLED was measured to be 76.42. H. Li *et al*. reported a CIE of (0.351, 0.376) and a high CRI of 85.6 for the WLED using two blue and red emissions^17^. However, the driving current to obtain white light was above 200 mA, which was very high compred to the typical current range for the operation of conventional LEDs. Moreover, white light was not monitored at relatively low driving current, resulting in the significant degradation in the CIE and the CRI. The CIE and CRI values of the WLED in our work using three primary colors were stably measured at the typical current ranges. As mentioned ealier, the IQE of our WLED was 47.43%, which is much higher than that of the ref. 17. The better IQE is also related to the contribution of three primary peaks to white light. Notably, white light was realized using InGaN-based QW structures without any phosphor materials or nanostructures. The blue-shifts in the blue, green, and red peak emission wavelengths with the increasing injection current from 10 to 120 mA were measured to be 2, 4, and 40.4 nm, respectively. These shifts were attributed to the band-filling effect. In addition, the increase in the number of carriers contributed to the compensation of the QCSE via Coulomb screening with the increasing the current, resulting in further blue-shifts in the emission wavelengths. However, the current-dependent blue-shift for the red emission is larger than those of the blue and green emissions, which is related to the relatively broad distribution of localized states. Specifically, the large piezoelectric field caused by the high compressive strain in the red QWs with the high In content favors the formation of In-rich clusters during the epitaxial growth of InGaN layers, resulting in a large distribution of localized energy states^14, 34^.

**Figure 6:**
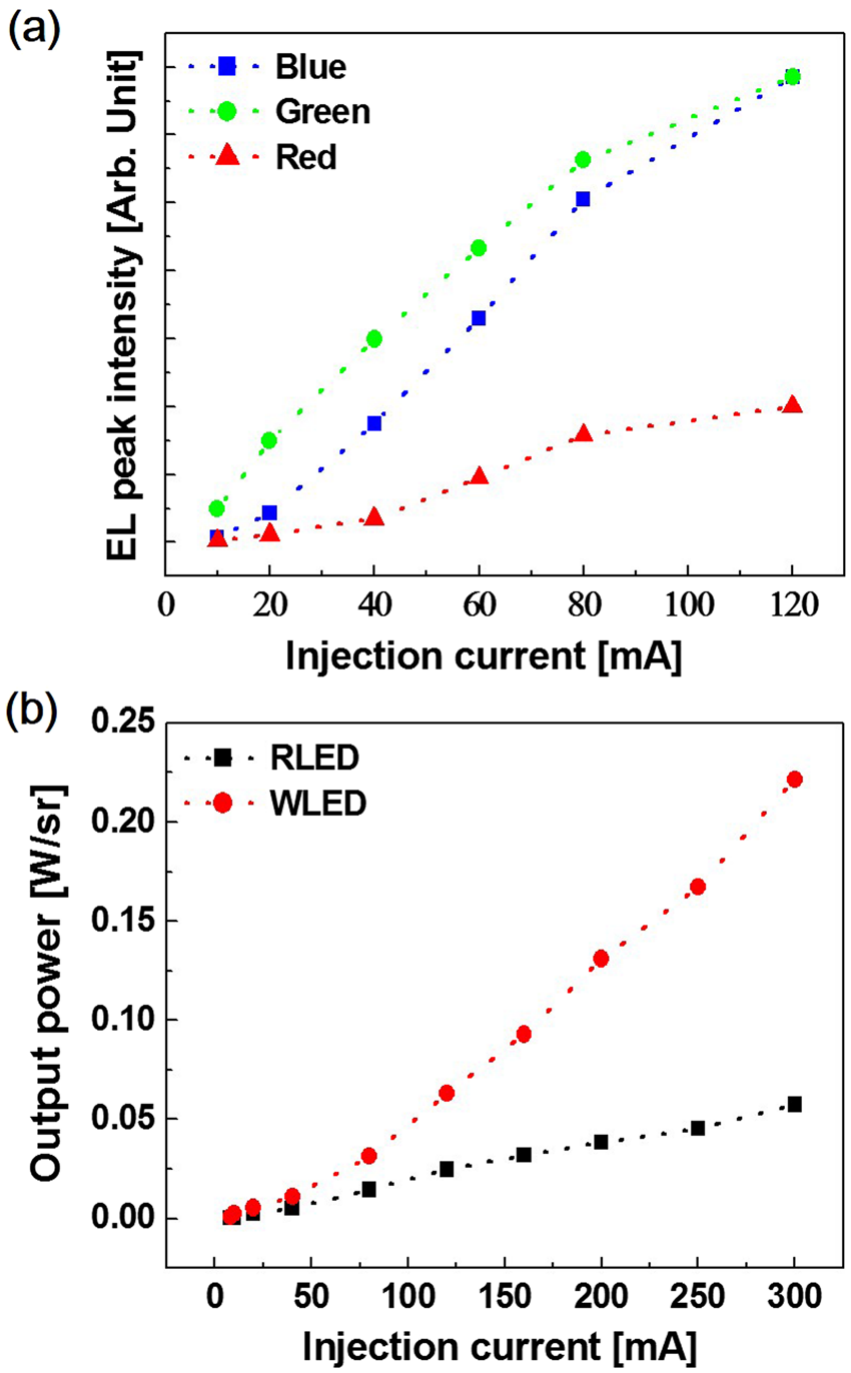
(**a**) Summary of the EL peak intensities for blue, green, and red emissions of the WLED as a function of the injection currents ranging from 10 to 120 mA, and (**b**) the output powers of the LED sample as a function of currents ranging from 8 to 300 mA, wherein the dotted lines are simply visual guides.

Figure 6(a) summarizes the EL intensities of the WLED sample as a function of the injection current ranging from 10 to 120 mA. The peak intensity of red emission is lower than those of the blue and green ones. In addition, the red emission increases less as a function of current than the other emissions. This can also be explained by the degradation in the QWs, which was largely caused by the difference in the optimal growth windows and the increase in the QCSE due to the compressive strain in the QWs. The output powers of the LED samples at currents ranging from 8 to 300 mA are summarized in Fig. 6(b). The output power of the WLED was 2.33 times stronger than that of the RLED at 120 mA (0.027 W/sr vs. 0.063 W/sr, respectively). Moreover, the WLED output power at 300 mA was 3.85 times stronger than that of the RLED sample. In the low current range, the output powers of the two LEDs were similar. However, with the increasing injection current, the WLED output power increased at a higher rate than that of the RLED, which can be explained by the increasing emission intensities of the blue and green spectra.

## Conclusion

In conclusion, we fabricated phosphor-free WLEDs with high color purity using InGaN-based MQWs emitting blue, green, and red. The red In_0.45_Ga_0.55_N/In_0.13_Ga_0.87_N QWs emitting 602 nm were realized by a new growth technique, namely, Ga-FI. The vertical and lateral distributions of the RLED and WLED optical emissions were analyzed using FM images. In the FM image obtained from the middle of the n-GaN cladding layer in the RLED, the shape of the light emission observed mostly around the LSPs exhibited a flower shape with six petals. The FM image of the WLED measured from the p-GaN cladding layer was light cyan because of the mixture of the three primary colors. In the EL spectra of the WLED at an injection current of 80 mA, bright white light was clearly exhibited, with three emission peaks positioned at 448, 510, and 597 nm. The CIE chromaticity coordinates of the EL spectrum for the WLED at an injection current of 80 mA was (0.316, 0.312), which is close to that of an ideal white-light source. Most phosphor-free white-light-emitting devices are based on nanostructures; thus, obtaining white light with three independent wavelengths using monolithically grown InGaN-based MQWs is a much more facile approach, thus representing a highly significant development in the ongoing research on WLEDs.

## Methods

The LED samples used in this study were grown on PSSs with LSPs by a MOCVD system. Trimethylgallium, trimethylindium, and ammonia were used as the Ga, In, and N sources, respectively. Disilane (Si_2_H_6_) and bis-cyclopentadienyl magnesium (CP_2_Mg) were used as n- and p-type doping sources, respectively. The LED structures consisted of a 2 μm-thick GaN buffer layer, a 3 μm-thick n-GaN layer, InGaN-based MQWs, and a 150 nm-thick p-GaN cladding layer. Figure 1(a) shows the schematic structure of the In_0.45_Ga_0.55_N/In_0.13_Ga_0.87_N-QW RLED. The In_0.45_Ga_0.55_N well was formed by the Ga-FI technique, wherein the Ga flow was interrupted for 2 seconds every 7 seconds during the deposition of In_0.3_Ga_0.7_N. Five Ga-FI deposition cycles were used for a single In_0.45_Ga_0.55_N well. To alleviate the well-known QCSE for the red QWs, 17 nm-thick In_0.13_Ga_0.87_N layers with relatively low In contents were used as barriers. The growth temperatures of the In_0.45_Ga_0.55_N well and the In_0.13_Ga_0.87_N barrier for the RLED were 800 and 895 °C, respectively. Figure 1(b) schematically shows the structure of the WLED, including red-emitting In_0.45_Ga_0.55_N/In_0.13_Ga_0.87_N QWs. The active layer consisted of two stacked In_0.18_Ga_0.82_N/GaN QWs, three stacked In_0.26_Ga_0.74_N/GaN QWs, and five stacked In_0.45_Ga_0.55_N/In_0.13_Ga_0.87_N QWs, corresponding to blue, green, and red emissions, respectively. For the blue and green InGaN/GaN MQWs, InGaN was deposited using the conventional growth mode.

The structural properties of the LED samples were measured using a STEM (JEM-2100F, JEOL Ltd.). In order to assess to the local carrier diffusion, surface inhomogeneity, and defect distribution in the LED samples, we evaluated FM images of the InGaN-based LEDs by changing FPs. For the FM measurements, a Hg lamp with a wavelength of 405 nm was used as an excitation source. The fluorescent images were obtained with a complementary metal–oxide–semiconductor camera. For the PL measurements, a He–Cd laser with a wavelength of 375 nm was used as the excitation source. The luminescence spectra were recorded using a CCD detector. The electrical properties of the LED samples were measured by increasing the injection current in a LED chip tester.

## Electronic supplementary material

Supplementary Information
