# Supplementary material for: Flower-Like Internal Emission Distribution of LEDs with Monolithic Integration of InGaN-based Quantum Wells Emitting Narrow Blue, Green, and Red Spectra
Source: Sci Rep. 2017 Aug 2;7:7164. doi: 10.1038/s41598-017-07808-2 (PMC5541069; doi:10.1038/s41598-017-07808-2)
Supplement: Supplementary file 1 — Supplementary Information [file 41598_2017_7808_MOESM1_ESM.doc]

Supplementary information

**Flower-Like Internal Emission Distribution of LEDs with Monolithic Integration of InGaN-based Quantum Wells Emitting Narrow Blue, Green, and Red Spectra**

**Kwanjae Lee^1,2+^, Ilgyu Choi^1+^, Cheul-Ro Lee^1^, Tae-Hoon Chung^2^, Yoon Seok Kim^3^, Kwang-Un Jeong^4^, Dong Chul Chung^5^, and Jin Soo Kim^1,*^**

^1^Division of Advanced Materials Engineering & Research Center of Advanced Materials Development, Chonbuk National University, Jeonju 54896, Republic of Korea

^2^Korea Photonics Technology Institute, Gwangju 61007, Korea, Republic of Korea

^3^Department of Nano-Optical Engineering, Korea Polytechnic University, Siheung 15073, Republic of Korea

^4^Department of Polymer-Nano Science and Technology, and Polymer Materials Fusion Research Centre, Chonbuk National University, Jeonju 54896, Republic of Korea

^5^Korea Institute of Carbon Convergence Technology, Jeonju 54853, Republic of Korea

^*^corresponding author. [kjinsoo@jbnu.ac.kr](mailto:kjinsoo@jbnu.ac.kr)

^+^these authors contributed equally to this work

**S1. PL spectra of the WLED measured at 10K and RT**


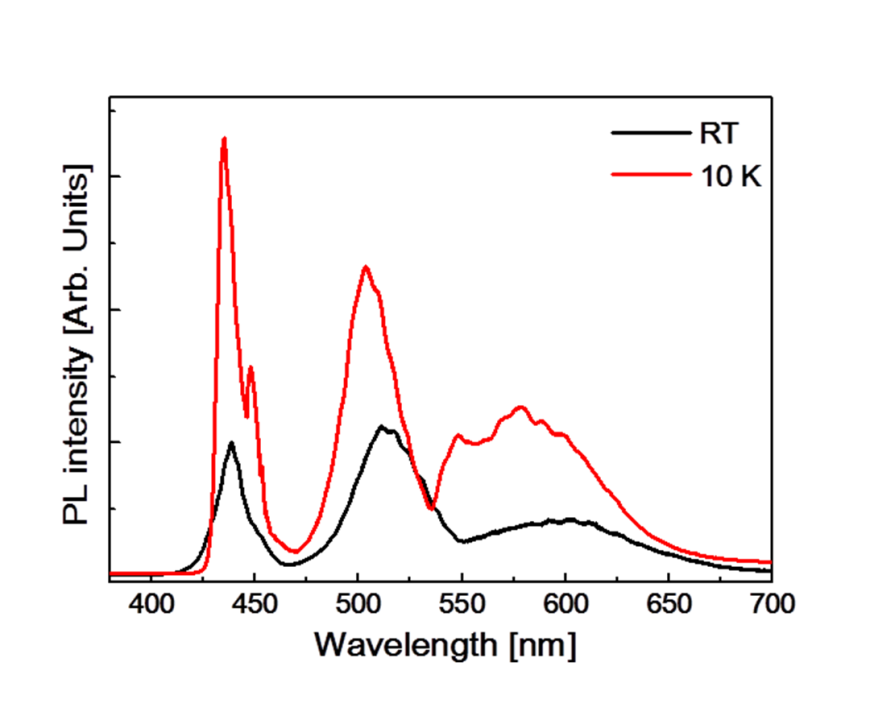


**Figure S1** | PL spectra of the WLED measured at 10 K and RT.

**S2. Flower-like internal emission distribution of LEDs with different wavelengths**


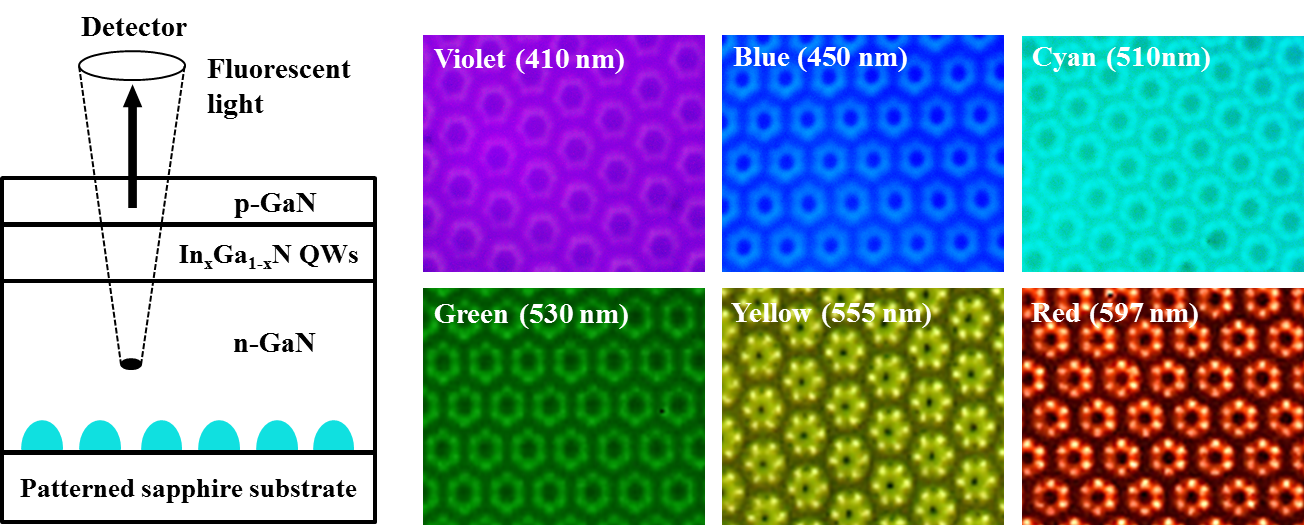


**Figure S2** | FM images measured at a focal point in the middle of the n-GaN cladding layer for the LED samples.
